# Prevalence and Recovery of Euthyroid Sick Syndrome in Pediatric Diabetic Ketoacidosis: A Retrospective Cohort Study

**DOI:** 10.3390/children13020296

**Published:** 2026-02-20

**Authors:** Youssef A. Alqahtani, Ayed A. Shati, Ayoub A. Alshaikh, Abdullah Saeed Mohammed Raffaa, Abdulaziz Saeed Alqahtani, Fahad Abdullah Saeed Alshahrani, Mohammed Fahad Nasser Alshahrani, Mohammed Abdulrahman Al-Sultan, Abdulaziz Saud Alotaibi, Yazeed Sultan Alshahrani, Ramy Mohamed Ghazy

**Affiliations:** 1 Department of Child Health, College of Medicine, King Khalid University, Abha 62421, Saudi Arabia; shatiayed@gmail.com; 2 Family and Community Medicine Department, College of Medicine, King Khalid University, Abha 61421, Saudi Arabia; alashaikh@kku.edu.sa; 3 College of Medicine, King Khalid University, Abha 61421, Saudi Arabia; abdul.raaff@hotmail.com (A.S.M.R.); abdulaziz111sq@gmail.com (A.S.A.); alsowadi6112@gmail.com (M.F.N.A.); mfn57@outlook.sa (M.A.A.-S.); documentsmy20@gmail.com (A.S.A.); ya.alshahrani2233@gmail.com (Y.S.A.); 4 Tropical Health Department, High Institute of Public Health, Alexandria University, Alexandria 21526, Egypt; 5 Health and Medical Research Centre, King Khalid University, Abha 61421, Saudi Arabia

**Keywords:** euthyroid sick syndrome, diabetic ketoacidosis, pediatrics, thyroid hormones, hospitalization, disease severity

## Abstract

**Highlights:**

**What are the main findings?**

- Euthyroid sick syndrome was diagnosed in 61.5% of pediatric patients with diabetic ketoacidosis, with two distinct hormonal phenotypes: isolated low FT3 and combined low FT4 and FT3.
- After 2 weeks, all children had at least one thyroid hormone returned to normal, but only 38.4% showed complete normalization of FT3, and 36.6% reached normal values for all thyroid parameters.

**What are the implications of the main findings?**

- Older age and higher baseline FT4 levels strongly predict complete thyroid recovery, highlighting key factors for early prognosis in pediatric ESS.
- Thyroid function tests during acute DKA should be interpreted cautiously, and follow-up testing is essential to avoid misdiagnosis or unnecessary hormone replacement.

**Abstract:**

**Background:** Euthyroid sick syndrome (ESS) is a common finding in critically ill patients, including children with diabetic ketoacidosis (DKA). However, its prevalence, specific hormonal patterns, and recovery in the pediatric population remain inadequately characterized. This study aimed to determine the prevalence of ESS in pediatric DKA, characterize its hormonal subtypes, and identify factors associated with short-term thyroid function recovery. **Methods:** A retrospective cohort study was conducted involving 182 pediatric patients (0–18 years) with type 1 diabetes mellitus admitted for DKA between January 2023 and June 2025. Thyroid function tests (TSH, FT4, FT3) were measured at presentations and two weeks after DKA resolution. ESS was defined using age-specific reference ranges. **Results:** The prevalence of ESS at DKA presentation was 61.5% (112/182). Two distinct hormonal phenotypes were identified: isolated low FT3 (n = 40, 35.7%) and combined low FT4 and FT3 (n = 72, 64.3%). Patients with the isolated low FT3 pattern were significantly younger (median 9.5 [3.50, 11.00] vs. 12.0 [8.50, 14.00] years, *p* = 0.004) and had milder hormonal derangement than the combined group. Normalization of FT4 was significantly lower in children with severe DKA compared with those with mild/moderate disease (50.0% vs. 84.8%, *p* = 0.002). FT3 normalization was also reduced in the severe group (20.0% vs. 42.4%), although this difference did not reach statistical significance (*p* = 0.078). After 2 weeks, all ESS patients (100%) had achieved normal levels of at least one thyroid hormone, with 38.4% reaching normalization of FT3 and 36.6% achieving normalization of all measured thyroid parameters. Age (adjusted odds ratio [aOR] = 2.08, 95% confidence interval (CI): 1.57–3.06, *p* < 0.001) and baseline FT4 level (aOR = 2.14, 95% CI: 1.51–3.32, *p* < 0.001) were positive predictors for complete recovery. **Conclusion:** ESS is highly prevalent in pediatric DKA, with distinct phenotypic patterns associated with age and the severity of acute illness, particularly the degree of acidosis. While transient in nature, complete biochemical recovery within two weeks is not universal. These findings underscore that thyroid function tests during acute DKA should be interpreted with caution to avoid misdiagnosis of primary thyroid disease, and they support the critical practice of follow-up testing after metabolic stabilization instead of immediate hormone replacement.

## 1. Introduction

Euthyroid sick syndrome (ESS), also called nonthyroidal illness syndrome or low triiodothyronine (T3) syndrome, describes the abnormal thyroid function test results often observed in critically ill patients in the medical intensive care unit (ICU) [1,2]. Although not a true syndrome, it reflects notable disruptions in the hypothalamic–pituitary–thyroid axis and occurs in around 75% of hospitalized individuals. It is commonly associated with severe illness, significant calorie deprivation, or major surgical procedures [3]. The typical hormonal pattern includes reduced total triiodothyronine (T3) and free triiodothyronine (FT3) levels, with free thyroxine (FT4) and thyroid-stimulating hormone (TSH) levels remaining low or within the normal range [4]. ESS arises because of severe metabolic stress and reflects the severity of the underlying illness rather than primary thyroid dysfunction [5]. The proposed mechanisms for the development of the syndrome include an imbalance in the activities of type I and type II deiodinases, diminished sensitivity of the hypothalamus and pituitary gland to thyroid hormones, and reduced protein binding and cellular uptake of T4 [6]. ESS has been observed in conditions such as liver disease, cardiac failure, renal failure, postoperative or stress states, frailty in elderly patients, malnutrition, and various malignancies [6,7,8].

Type 1 diabetes mellitus (T1DM) is a prevalent autoimmune disease that is frequently associated with other autoimmune disorders, including celiac disease and autoimmune hypothyroidism [9]. The clinical presentation of T1DM varies widely, ranging from mild symptoms to diabetic ketoacidosis (DKA), which occurs in approximately 30–50% of children at the time of initial diagnosis [10,11]. T1DM, particularly during episodes of DKA, is associated with thyroid hormone alterations characteristic of ESS [12]. It has been reported that roughly 24.5% to 57% of children experiencing DKA show signs of ESS [13]. In patients with T1DM, poor glycemic control predisposes them to DKA. Therefore, the presence of ESS serves as a marker of disease severity and poor prognosis [14,15]. Several studies found that children with T1DM who presented with both DKA and ESS had higher glycated hemoglobin (HbA1c) levels, higher anion gaps, and elevated plasma glucose, along with lower bicarbonate levels, compared with those who did not have ESS [14,16]. They also tend to experience a slower recovery [13]. Management of recovery-phase ESS is directed at the underlying illness. Although few studies have examined factors influencing recovery from critical illness beyond nutrition, evidence does exist. A large trial demonstrated that postponing supplemental parenteral nutrition until day 8 in ICU patients resulted in fewer complications and more rapid recovery compared with initiating parenteral nutrition within 48 h [17]. In a sub-analysis involving patients with ESS, late feeding likewise was associated with improved recovery and lower complication rates—but it also accentuated the characteristic ESS-associated alterations in thyroid hormones (TSH, total T4, T3, and T3:rT3 ratio), whereas early feeding attenuated those hormonal changes [18]. We therefore hypothesize that ESS is highly prevalent among pediatric patients with DKA and that specific clinical and biochemical characteristics at presentation, including markers of illness severity, predict the likelihood and rate of early ESS recovery. Furthermore, we evaluated the recovery rate of ESS two weeks after resolution of DKA to characterize early recovery. Although thyroid hormone abnormalities induced by acute metabolic stress begin to improve within days after correcting acidosis and insulin deficiency, complete normalization of peripheral thyroid levels and full restoration of the hypothalamic–pituitary–thyroid axis may require 6–8 weeks, particularly in more severe cases [18,19]. The two-week follow-up was therefore selected to capture initial recovery trends while minimizing the risk of misinterpreting transient hormonal disturbances as persistent thyroid disease [20].

## 2. Materials and Methods

### 2.1. Study Design

We conducted a retrospective cohort study of pediatric patients admitted with DKA between 1 January 2023 and 30 June 2025 at King Khalid University Medical City and Abha Maternity and Children Hospital. Baseline thyroid function tests were obtained at the time of DKA presentation. Follow-up thyroid hormone levels were extracted from medical records two weeks after admission to assess the point prevalence of ESS and patterns of early recovery. Recovery from ESS was defined as normalization of FT3 levels to age-specific reference ranges after two weeks of DKA treatment.

### 2.2. Study Population and Sample Size

The study included pediatric patients aged up to 18 years with a confirmed diagnosis of T1DM who presented with DKA during the study period. DKA was defined according to standard biochemical criteria [21]. Eligible participants were required to have thyroid function tests, including TSH, FT3, and FT4, performed at the time of DKA presentation and repeated two weeks after resolution of DKA. Patients were excluded if they had a known diagnosis of thyroid disease prior to admission or were receiving medications known to affect thyroid function. Thyroid function results at the two-week follow-up were analyzed to characterize early recovery patterns rather than to establish definitive thyroid diagnoses; therefore, persistent abnormalities at this time point were interpreted as incomplete early recovery and not as diagnostic of primary or central thyroid disease. Additional exclusion criteria included chronic systemic illnesses (e.g., chronic kidney disease or malignancy), non-diabetic causes of ketoacidosis, absence of thyroid function testing at follow-up, or a diagnosis of type 2 diabetes mellitus.

Although this was a retrospective study including all eligible patients, a sample size estimation was conducted to assess the adequacy of the cohort. Sample size estimation was performed using G*Power version 3.1.9.4 (Heinrich-Heine-Universität Düsseldorf, Düsseldorf, Germany). Based on previously reported ESS prevalence rates of 24.5% [22] and 37.3% [13], an effect size (Cohen’s g) of 0.128 was calculated using a binomial test framework. A two-sided exact test for proportions was applied; with a type I error rate (α) of 0.05 and a desired power of 95%. Assuming a constant reference proportion of 0.245, the analysis indicated that a total sample size of 172 participants was required to detect a statistically significant difference, yielding an actual power of 95%.

### 2.3. Study Variables

Demographic data collected included age, sex, weight, height, and body mass index (BMI), with age categorized into four groups: below 4, 5–9, 10–14, and 15–18 years. Biochemical variables included blood glucose at diagnosis, HbA1c (%), and baseline thyroid function tests (TSH, FT4, FT3), with follow-up thyroid hormone measurements obtained approximately two weeks after DKA resolution. Glycemic control was assessed using HbA1c and classified as good (<7%), moderate (7–8.9%), or poor (≥9%).

### 2.4. Study Outcomes

Primary outcome: Point prevalence of ESS and its types among children diagnosed with DKA.

Secondary outcomes: Thyroid function recovery after DKA management was defined as the normalization of laboratory values for FT3, FT4, and TSH. Partial normalization was defined as the clinical scenario where thyroid hormone parameters (TSH/FT3/FT4) returned to their age-appropriate normal ranges following resolution of DKA in patients with ESS.

### 2.5. Data Collection

Demographic, clinical, and biochemical data at the time of DKA presentation were systematically extracted from patient records. Thyroid function tests were recorded both at DKA diagnosis and two weeks after DKA resolution. Age-specific pediatric reference ranges were applied to classify thyroid function accurately. Missing values were recorded as “NA” and were addressed through complete-case analysis, which means only the available data for each variable were utilized. The amount of missing data was minimal, and no imputation was carried out, thereby ensuring that the analyses accurately represent the observed data without the risk of bias from estimated values.

### 2.6. Operational Definitions

Thyroid function classification: Normal thyroid function was defined as TSH, free T4 (FT4), and free T3 (FT3) within age-specific reference ranges [22]:

- <1 year: TSH 0.8–8.2 mIU/L, FT4 11–23 pmol/L, FT3 5.5–10.0 pmol/L.
- 1–6 years: TSH 0.7–6.0 mIU/L, FT4 14–26 pmol/L, FT3 5.7–13.1 pmol/L.
- 7–12 years: TSH 0.6–5.0 mIU/L, FT4 13–23 pmol/L, FT3 4.5–10.0 pmol/L.
- ≥13 years: TSH 0.4–4.2 mIU/L, FT4 12–21 pmol/L, FT3 3.5–9.4 pmol/L.

ESS was defined as low FT3 for age, with TSH typically within the age-specific reference range (though it may be transiently altered). FT4 could be normal or low. ESS subcategories included:

- Low FT3: Isolated low FT3 with normal FT4
- Low FT3 and FT4: Low FT3 and low FT4

### 2.7. Ethical Approval

The study was conducted in accordance with the Declaration of Helsinki and national regulations on human research. Ethical approval was obtained from the Research Ethics Committee at King Khalid University (HAPO-06-B-001; KKU-171-2025-31). As this was a retrospective study, the requirement for written informed consent was waived. Patient confidentiality was ensured by de-identifying all records, and data were securely stored on password-protected institutional computers accessible only to the research team.

### 2.8. Statistical Analysis

All data were carefully checked for completeness, accuracy, and consistency prior to analysis. Normality was assessed using the Shapiro–Wilk test and visual inspection of histograms. Continuous variables were summarized as mean ± standard deviation (SD) for normally distributed data or median (interquartile range, IQR) for non-normally distributed data. Between two independent non-normally distributed groups, the Mann–Whitney U test was performed. Categorical variables were summarized as frequencies and percentages and compared using Chi-square tests or Fisher’s exact tests, as appropriate. The relationships among continuous variables were evaluated using Pearson correlation coefficients (r) to determine linear associations between thyroid hormone levels and various demographic or biochemical factors. The strength of these correlations was categorized based on conventional thresholds: weak (|r| = 0.10–0.29), moderate (|r| = 0.30–0.49), and strong (|r| ≥ 0.50). Variables showing significance in bivariate analyses were subsequently included in multivariable logistic regression models to identify independent predictors of ESS recovery. Adjusted odds ratios (aOR) along with 95% confidence intervals (CI) were provided for each predictor. The model’s fit and explanatory capability were evaluated using Pseudo R^2^ and the Akaike Information Criterion (AIC). To visualize data, the heatmap showcasing the distributions and relationships of key variables was generated using the ggplot2 package. All statistical analyses were performed using R version 4.3 (R Foundation for Statistical Computing, Vienna, Austria), with relevant packages including dplyr, tidyr, janitor, and ggplot2.

## 3. Results

The study involved 182 children and adolescents with an average age of 11.3 ± 4.3 years, nearly half (46.2%) of whom were aged between 10 and 14 years. Among the participants, 53.8% were male. The mean BMI was 18.8 ± 3.1 kg/m^2^. When classified using age- and sex-specific percentiles, most participants (67.0%) were considered normal weight, while 8.8% were underweight and 15.4% were overweight. Additionally, 8.8% were classified as obese. At presentation, mean blood glucose was 482.7 ± 68.5 mg/dL, and mean HbA1c was 11.5 ± 1.5%. Most patients (96.4%) had poor glycemic control (HbA1c ≥ 9%). Severe acidosis was observed in 17.9% of cases. Overall, ESS was identified in 112 patients, corresponding to a prevalence of 61.5% at the time of DKA presentation, as shown in Table 1.

Table 2 summarizes the two distinct hormonal phenotypes observed among children with ESS. In the isolated low FT3 group (N = 40, 35.7%), all patients exhibited reduced FT3 levels with preserved FT4 and normal TSH concentrations. In contrast, the combined low FT4 and FT3 group (N = 72, 64.3%) demonstrated a more pronounced degree of hormonal suppression, characterized by concurrent reductions in both FT3 and FT4, while TSH levels remained within the normal range in all patients. Children with isolated low FT3 were significantly younger than those with combined low FT4 and FT3 (median 9.5 [3.50, 11.00] vs. 12.0 [8.50, 14.00] years, *p* = 0.004) and had lower median body weight (26.00 [15.00, 35.00] vs. 33.50 [27.00, 45.50] kg, *p* = 0.001). Baseline FT3 levels were significantly higher in the isolated low FT3 group (2.8 [2.4, 4.0] vs. 2.3 [1.9, 2.8] pmol/L, *p* = 0.001), and this difference persisted at the two-week follow-up (4.80 [4.20, 5.10] vs. 4.00 [3.10, 4.30] pmol/L, *p* < 0.001). Despite these differences, the magnitude of FT3 change after 2 weeks of DKA recovery did not differ significantly between groups (1.30 [0.80, 2.10] vs. 0.90 [0.55, 2.30] pmol/L, *p* = 0.40). Additional hormonal differences were observed at presentation: children with combined low FT4 and FT3 had significantly lower FT4 concentrations (11.0 [10.4, 12.0] vs. 14.6 [14.0, 15.7] pmol/L, *p* < 0.001) and lower TSH levels (1.2 [0.9, 2.4] vs. 2.3 [1.6, 3.0] mIU/L, *p* < 0.001). Baseline glucose levels were modestly but significantly higher in the isolated low FT3 group (502.0 [444.0, 571.5] vs. 462.0 [430.0, 518.5] mg/dL, *p* = 0.040), whereas HbA1c values and sex distribution did not differ significantly between groups.

**Figure 1:**
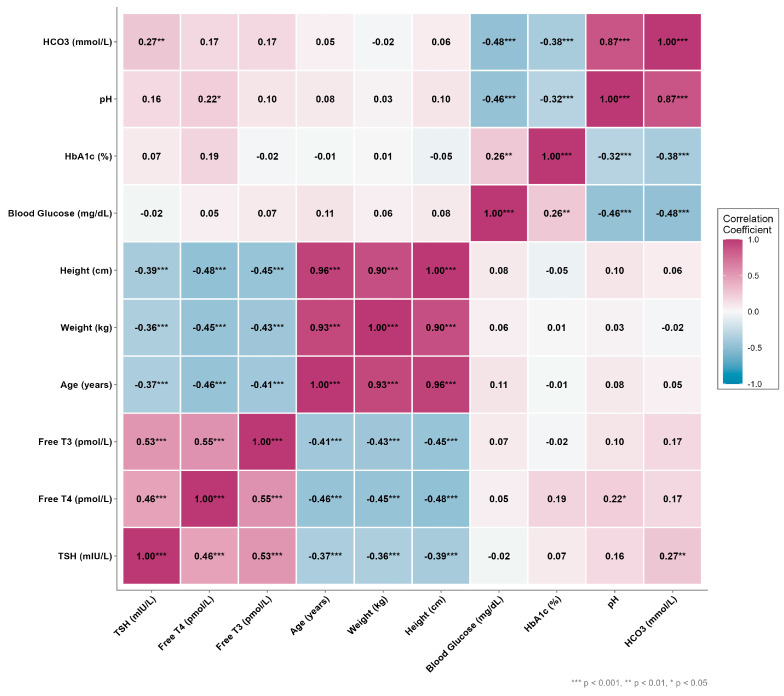
Correlations of thyroid hormones with anthropometric and acid–base parameters in pediatric euthyroid sick syndrome cases. All laboratory measurements were obtained at presentations with diabetic ketoacidosis (DKA), except follow-up FT3 and FT3 change, which reflect recovery-phase assessments.

Free T3 was inversely correlated with height (r = −0.447, *p* < 0.001), weight (r = −0.427, *p* < 0.001), and age (r = −0.413, *p* < 0.001). Free T4 also demonstrated negative correlation with height (r = −0.482, *p* < 0.001), weight (r = −0.453, *p* < 0.001), and age (r = −0.457, *p* < 0.001), and showed a weak positive correlation with pH (r = 0.222, *p* = 0.019). TSH was negatively correlated with height (r = −0.387, *p* < 0.001), weight (r = −0.361, *p* < 0.001), and age (r = −0.369, *p* < 0.001) and exhibited a weak positive correlation with HCO3 (r = 0.270, *p* = 0.004). Figure 1

Summary statistics showed that among 112 patients with low FT3, 43 (38.4%) achieved normalization within two weeks, 36.6% achieved normalization of all parameters, and 100% achieved normalization of either FT3 or FT4. Pattern-specific analysis revealed higher normalization rates in patients with isolated low FT3 (19/40, 47.5%) compared to those with combined low FT4 and FT3 (24/72, 33.3%). However, this difference was not statistically significant (*p* = 0.2). At baseline, median TSH was 1.75 mIU/L [1.10–2.90], which increased to 2.20 mIU/L [1.55–3.10] at follow-up, representing a median absolute change of 0.40 mIU/L [0.20–0.75] and a median percentage increase of 25.2% [6.6–58.6%]. Baseline FT4 was 12.00 pmol/L [10.65–14.05], rising to 13.90 pmol/L [12.70–14.90] at follow-up, with a median change of 1.35 pmol/L [0.60–2.30] and a 10.6% [4.3–21.6%] increase. FT3, the most sensitive marker of peripheral thyroid metabolism, increased from 2.40 pmol/L [1.95–3.40] to 4.10 pmol/L [3.40–4.85], with a median absolute change of 1.20 pmol/L [0.60–2.15] and a median percentage increase of 46.3% [23.9–93.7%]. At follow-up, TSH was normal in all 112 patients (100%), and FT4 was normalized in 88 patients (78.6%), as shown in Table 3.

Children presenting with severe DKA showed significantly poorer recovery of thyroid function compared to those with mild or moderate DKA. FT4 normalization was markedly lower in the severe group (50.0% vs. 84.8%, *p* = 0.002), and the improvement in FT3 levels was also reduced, with only 20.0% achieving normalization compared to 42.4% in the mild/moderate group (*p* = 0.078). Additionally, biochemical recovery was slower in severe DKA, reflected by smaller increases in FT3 (*p* = 0.039) despite larger FT4 changes (*p* = 0.035). Children with severe DKA also had significantly higher glucose levels at presentation (*p* < 0.001), as shown in Table 4.

**Figure 2:**
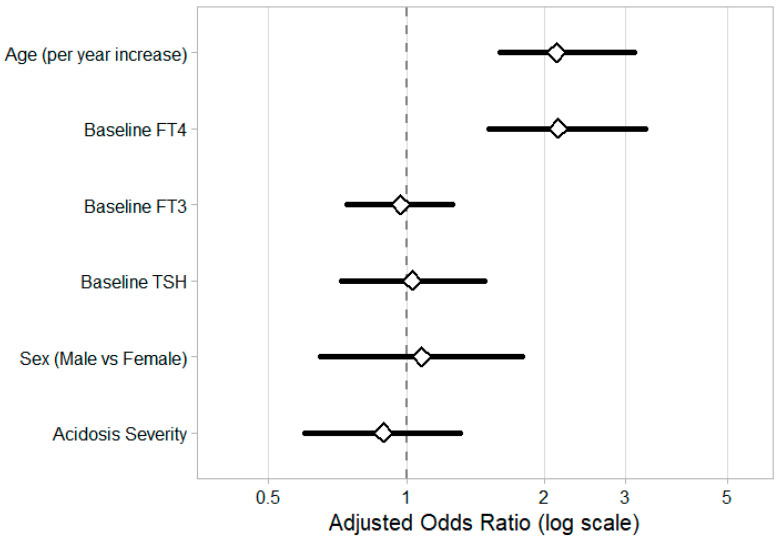
Predictors of complete thyroid recovery in ESS Patients: impact of age and baseline thyroid patterns.

The logistic regression model used to identify predictors of complete recovery in patients with ESS showed a good overall fit, evidenced by a Pseudo R^2^ (McFadden) of 0.48, a reduction in deviance from 147.1 to 77.0, and an AIC of 90.95. These statistics suggest that the variables analyzed—age, sex, baseline TSH, FT4, FT3, and acidosis severity—accounted for a significant amount of the variability in recovery outcomes. Age emerged as a strong positive predictor, with each additional year more than doubling the odds of achieving complete recovery (aOR = 2.12, 95% CI: 1.60–3.14, *p* < 0.001). Likewise, baseline FT4 levels were positively correlated with recovery (aOR = 2.14, 95% CI: 1.51–3.32, *p* < 0.001). Conversely, sex, baseline TSH and FT3 levels, and acidosis severity did not show statistically significant predictive value (*p* > 0.05). Figure 2 presents a forest plot that visually summarizes the odds ratios and 95% confidence intervals for all predictors.

## 4. Discussion

Summary of the main study findings: This retrospective cohort study investigated the point prevalence, hormonal patterns, and early recovery of ESS among 182 pediatric patients presenting with DKA between January 2023 and June 2025. The study found that ESS was highly prevalent, affecting nearly three-fifths of children at the time of DKA presentation. Two distinct hormonal subtypes were identified: isolated low FT3 and combined low FT3 and low FT4. Thyroid follow-up tests conducted about two weeks after the resolution of DKA indicated that 38.4% of patients with ESS had normalized their FT3 levels, with a slightly higher—though not statistically significant—recovery rate observed in the isolated low FT3 group. Additionally, 36.6% of patients achieved normalization of all thyroid parameters, while 100% attained normal levels of either FT3 or FT4.

### 4.1. Interpretation of the Main Study Findings

Prevalence of ESS among DKA: In the current study, the prevalence of ESS among pediatric patients with DKA was 61.5%. A similar prevalence of 57.8% has been reported in a previous study involving adult patients with type 1 or type 2 diabetes who presented with DKA or diabetic ketosis [23]. On the other hand, a lower ESS prevalence of 24.5% was reported in a cohort of 208 newly diagnosed patients with T1DM [22]. In another study of 161 participants, 60 (37.3%) were found to have ESS [13]. ESS in DKA likely results from acute metabolic stress altering deiodinase activity, cytokine-mediated suppression of the hypothalamic–pituitary axis, and reduced T4-to-T3 conversion. These results indicate that ESS occurs fairly frequently in the setting of DKA. This pattern may be related to the acute physiological stress and metabolic disturbances accompanying DKA. Moreover, variation in study cohorts, age distributions, and diagnostic definitions contributes to the differences in reported prevalence among studies. Taken together, these findings underscore the need to screen for ESS in pediatric patients with DKA, as early detection may influence both treatment decisions and clinical outcomes.

Pattern of ESS: The reported findings identified two distinct hormonal patterns of ESS in children with DKA: one with isolated low FT3 and another with both low FT3 and low FT4, each occurring despite normal TSH levels. This differentiation is both clinically and physiologically important for several reasons. First, the consistently normal TSH observed in all 112 ESS cases provides convincing evidence favoring ESS over primary thyroid disease. In primary hypothyroidism, TSH is usually elevated, whereas in central hypothyroidism, it tends to be inappropriately low or normal. In contrast, the presence of normal TSH alongside reduced FT3 (with or without reduced FT4) is characteristic of ESS in the context of acute systemic illness [24]. This pattern suggests the thyroid axis disturbance is an adaptive, reversible response rather than true thyroid disease. Recognizing these subtypes helps clinicians avoid misdiagnosis (preventing unnecessary thyroid hormone therapy or endocrine workup), stratify severity (the combined low FT3/FT4 group may indicate greater metabolic derangement and need for closer monitoring), and guide retesting (repeat thyroid function after clinical recovery, as in ~2 weeks post-DKA, since both subtypes should resolve spontaneously).

Glycemic parameters and ESS: In this group of children with DKA, HbA1c levels were similarly high across different phenotypes of ESS, indicating that the degree of chronic poor glycemic control was comparable among them. However, baseline blood glucose levels varied significantly between the groups. The lack of variation in HbA1c suggests that the phenotype of ESS is not linked to long-term hyperglycemia, since HbA1c reflects average glucose levels over recent weeks to months and does not account for acute metabolic changes. In contrast, the observation that patients with isolated low FT3 had higher initial glucose levels compared to those with both low FT4 and FT3 suggests that acute hyperglycemia does not predict the degree of thyroid hormone suppression. This separation of findings supports the idea that the severity of ESS during DKA is primarily influenced by the overall stress of acute systemic factors—including the release of inflammatory cytokines, excess counter-regulatory hormones, caloric restriction, and impaired peripheral conversion of T4 to T3—rather than by absolute glucose levels or long-term glycemic control [25,26].

DKA severity and recovery: The normalization of FT4 levels was significantly lower, and the recovery of FT3 was diminished, indicating a greater suppression of the hypothalamic–pituitary–thyroid axis and a delay in the peripheral conversion of T4 to T3 among children with severe acidosis. Similar observations were made in adults aged 18–36 [20]. Notably, although there were greater absolute increases in FT4 levels in the severe DKA group, post-recovery FT4 levels often remained below the normal range, while improvements in FT3 lagged, suggesting a delayed biochemical recovery [20,27]. While bivariate analyses indicated that the severity of DKA has a strong influence on thyroid recovery, multivariable analysis did not identify it as an independent predictor, likely due to collinearity with baseline FT4 levels or a limited sample size in the severe subgroup. These results underscore the importance of careful interpretation of thyroid function tests following DKA, the necessity for longer follow-up in severe cases, and the need to avoid unnecessary thyroid hormone treatments.

ESS recovery rate: In this study, 38.4% of children with ESS showed recovery of FT3 levels after two weeks, while 36.6% normalized all parameters. Additionally, 100% of the participants achieved normalization of either FT3 or FT4, suggesting that thyroid-axis suppression in pediatric DKA is reversible, albeit at varying rates of resolution. Age emerged as a significant predictor of recovery from ESS, particularly in the context of DKA. Research indicates that older children tend to recover thyroid function more readily than their younger counterparts. This phenomenon may stem from developmental differences; younger children often exhibit a more pronounced or prolonged suppression of the hypothalamic–pituitary–thyroid axis when faced with metabolic stress. Various physiological factors could contribute to this disparity, including differences in thyroid hormone metabolism, deiodinase enzyme activity, and the responsiveness of stress hormones [28]. Clinically, these findings underscore the importance of considering a child’s age when interpreting thyroid function tests. In younger patients, persistent abnormalities are more likely indicative of a delayed biochemical recovery from ESS rather than an intrinsic thyroid disorder. Therefore, careful monitoring and appropriate follow-up intervals are essential to distinguish between temporary hormonal adjustments and underlying thyroid dysfunction in this vulnerable population. In addition to age, baseline FT4 levels were also identified as a significant predictor of recovery. Similarly, a prospective study of 70 critically ill children in the pediatric ICU reported that low FT4 was more common in non-survivors than survivors (50% vs. 19.2%, *p* = 0.028), highlighting its potential prognostic value [29].

In the present study, a substantial proportion of children achieved normalization of thyroid hormones within two weeks following DKA resolution. In the same vein, a study conducted by Yang et al. [30] showed that abnormalities in thyroid hormone levels during acute illness normalized after 10 days of therapy. A Turkish study reported even faster recovery, with 50% of children normalizing their thyroid function within the first 7 days and 86.6% by days 7–14; importantly, 90% of patients reassessed between days 15 and 90 showed full recovery, and all children re-evaluated after day 91 had complete normalization [22]. Moreover, Xiang et al. [31] further confirmed that thyroid alterations associated with ketoacidosis reverse following correction of the metabolic disturbance. These findings collectively support the concept that ESS in pediatric DKA is a stress-related, reversible phenomenon. Thyroid function tests obtained during DKA or in the early post-DKA period should therefore be interpreted with caution. Persistent abnormalities beyond two weeks likely reflect delayed biochemical recovery rather than true thyroid disease. We recommend repeat testing at 6–8 weeks for children whose thyroid values remain abnormal and further evaluation for primary autoimmune thyroid disease or central hypothyroidism if dysfunction persists beyond three months, as ongoing abnormalities at this stage are unlikely to be explained by ESS [1]

### 4.2. Implication of This Study

Identifying ESS in children presenting with DKA offers valuable clinical insights. In our cohort, we found that the severity of acidosis (as determined by bivariate analysis), along with age and baseline free T4 levels (from multivariable analysis), was associated with delays in thyroid recovery. This aligns with previous studies suggesting that ESS may correlate with more severe clinical presentations and prolonged hospital stays, indicating its potential as a marker of illness severity rather than indicating a primary thyroid issue [32]. Consequently, variations in thyroid function observed during acute DKA should be interpreted with caution, and thyroid hormone replacement is generally not required unless dysfunction persists beyond metabolic stabilization, typically after 8 to 12 weeks. Follow-up evaluations, particularly after two weeks and between six and eight weeks, are advised to verify normalization. Additional research is needed to explore whether targeted interventions can improve recovery and clinical outcomes in this high-risk pediatric group.

### 4.3. Limitations and Strengths

This study’s strengths include the application of age-specific pediatric reference ranges for diagnosing ESS, a substantial cohort size, and the use of robust statistical methods. However, several limitations must be considered. The retrospective, single-center design introduces potential selection bias and limits the generalizability of our findings. Furthermore, the absence of data on key confounding variables—such as nutritional status, the use of specific medications (e.g., glucocorticoids), inflammatory markers (e.g., C-reactive protein), and detailed fluid resuscitation protocols—means that residual confounding may influence the observed associations. The relatively short, two-week follow-up period, while informative for early recovery, may be insufficient to capture the full normalization of thyroid function in all patients, particularly those with severe ESS.

## 5. Conclusions

In conclusion, this study confirms that ESS is highly prevalent, complicating the initial presentation in more than three-fifths of pediatric DKA cases. We identified two distinct biochemical phenotypes—isolated low FT3 and combined low FT4 and FT3—with the latter associated with greater metabolic derangement and older age. Critically, the syndrome is largely transient, with significant hormonal improvement observed within two weeks. However, full biochemical recovery is not universal within this short timeframe and is strongly modulated by age and baseline FT4 levels, highlighting the importance of individualized follow-up in patients with severe or persistent thyroid hormone suppression.

## Figures and Tables

**Table 1:** Descriptive Characteristics of Study Participants (N = 182).

Variable	Level/Statistic	Overall (N = 182)
Age (years)	Mean ± SD	11.3 ± 4.3
	4 years or less	22 (12.1%)
	5–9 years	28 (15.4%)
	10–14 years	84 (46.2%)
	15–18 years	48 (26.4%)
Sex	Female	84 (46.2%)
	Male	98 (53.8%)
BMI (kg/m^2^)	Mean ± SD	18.8 ± 3.1
	Underweight (<5th percentile)	16 (8.8%)
	Normal weight (5th–84th percentile)	122 (67.0%)
	Overweight (85th–94th percentile)	28 (15.4%)
	Obese (≥95th percentile)	16 (8.8%)
Blood glucose	Mean ± SD	482.7 ± 68.5
HbA1C	Mean ± SD	11.5 ± 1.5
	Moderate (HbA1c 7–8.9%),	4 (3.6%)
	Poor (HbA1c ≥ 9%)	108 (96.4%)
Acidosis severity	Mild/Moderate	92 (82.1%)
	Severe	20 (17.9%)
Euthyroid sick syndrome		112 (61.5%)

BMI, body mass index; HbA1c, glycated hemoglobin. Continuous variables are presented as mean ± standard deviation (SD), and categorical variables as number (percentage). BMI categories were defined using age- and sex-specific percentiles according to Centers for Disease Control and Prevention (CDC) growth charts. Euthyroid sick syndrome (ESS) was defined as low free triiodothyronine (FT3) levels in the presence of normal or low thyroid-stimulating hormone (TSH) and free thyroxine (FT4) at the time of DKA presentation. Percentages may not total 100% because of rounding.

**Table 2:** Clinical, biochemical, and hormonal characteristics according to ESS phenotype.

Variable	Isolated Low FT3 (N = 40)	Low FT4 and FT3 (N = 72)	*p*-Value
Hormonal pattern at presentation			
TSH (normal), n (%)	40 (100%)	72 (100%)	—
Free T4 (normal), n (%)	40 (100%)	0 (0%)	—
Free T4 (low), n (%)	0 (0%)	72 (100%)	—
Free T3 (low), n (%)	40 (100%)	72 (100%)	—
Demographic characteristics			
Age (years), median [Q1, Q3]	9.50 [3.50, 11.00]	12.00 [8.50, 14.00]	0.004
Female sex, n (%)	14 (35.0%)	34 (47.2%)	0.30
Male sex, n (%)	26 (65.0%)	38 (52.8%)	
Weight (kg), median [Q1, Q3]	26.00 [15.00, 35.00]	33.50 [27.00, 45.50]	0.001
Glycemic parameters			
HbA1c (%), median [Q1, Q3]	12.0 [10.4, 12.6]	11.3 [10.3, 12.4]	0.20
Baseline glucose (mg/dL), median [Q1, Q3]	502.0 [444.0, 571.5]	462.0 [430.0, 518.5]	0.04
Thyroid function at presentation			
TSH (mIU/L), median [Q1, Q3]	2.3 [1.6, 3.0]	1.2 [0.9, 2.4]	<0.001
FT4 (pmol/L), median [Q1, Q3]	14.6 [14.0, 15.7]	11.0 [10.4, 12.0]	<0.001
FT3 (pmol/L), median [Q1, Q3]	2.8 [2.4, 4.0]	2.3 [1.9, 2.8]	0.001
Follow-up thyroid parameters (2 weeks)			
Follow-up FT3 (pmol/L), median [Q1, Q3]	4.80 [4.20, 5.10]	4.00 [3.10, 4.30]	<0.001
FT3 change (pmol/L), median [Q1, Q3]	1.30 [0.80, 2.10]	0.90 [0.55, 2.30]	0.40

ESS: Euthyroid sick syndrome (also known as non-thyroidal illness syndrome). FT3: free triiodothyronine, FT4: free thyroxine, TSH: thyroid-stimulating hormone. Data are presented as frequency (percentage). The isolated low FT3 pattern refers to patients with normal TSH and FT4 but low FT3; the low FT4 and FT3 pattern refers to patients with normal TSH but low FT4 and FT3. All patterns were measured during acute diabetic ketoacidosis (DKA) presentation.

**Table 3:** Thyroid hormone recovery outcomes and biochemical changes at follow-up (N = 112).

Parameter	Overall (N = 112)	Isolated Low FT3 (N = 40)	Low FT4 and FT3 (N = 72)	Baseline Median [Q1, Q3]	Follow-up Median [Q1, Q3]	Absolute Change Median [Q1, Q3]	% Change Median [Q1, Q3]
TSH remained normal, n (%)	112 (100.0%)	40 (100.0%)	72 (100.0%)	1.75 [1.10, 2.90]	2.20 [1.55, 3.10]	0.40 [0.20, 0.75]	25.23 [6.57, 58.61]
FT4 normalized, n (%)	88 (78.6%)	40 (100.0%)	48 (66.7%)	12.00 [10.65, 14.05]	13.90 [12.70, 14.90]	1.35 [0.60, 2.30]	10.64 [4.26, 21.60]
FT3 normalized, n (%)	43 (38.4%)	19 (47.5%)	24 (33.3%)	2.40 [1.95, 3.40]	4.10 [3.40, 4.85]	1.20 [0.60, 2.15]	46.30 [23.86, 93.66]
Any parameter normalized, n (%)	112 (100.0%)	40 (100.0%)	72 (100.0%)	—	—	—	—
All parameters normalized, n (%)	41 (36.6%)	19 (47.5%)	22 (30.6%)	—	—	—	—

ESS, euthyroid sick syndrome; FT3, free triiodothyronine; FT4, free thyroxine; TSH, thyroid-stimulating hormone; HbA1c, hemoglobin A1c. Continuous variables are presented as median [first quartile (Q1), third quartile (Q3)], and categorical variables as frequency (percentage). *p* values were calculated using the Mann–Whitney U test for continuous variables and the chi-square test for categorical variables, with statistical significance defined as *p* < 0.05. Normalization was defined as return to age-appropriate reference ranges after resolution of acute illness, and values are reported as the frequency (percentage) of patients achieving normalization. All patients maintained normal TSH levels at follow-up. The isolated low FT3 pattern demonstrated higher FT4 and FT3 normalization rates compared with the combined low FT4 and FT3 pattern, suggesting a differential recovery trajectory related to the severity of baseline thyroid dysfunction.

**Table 4:** DKA severity (mild/moderate vs. severe) and thyroid recovery outcomes (N = 112).

Variable	Mild/Moderate DKA (n = 92)	Severe DKA (n = 20)	*p*-Value
TSH at diagnosis (mIU/L)	2.0 [1.1, 2.9]	1.6 [0.9, 2.4]	0.3
FT4 at diagnosis (pmol/L)	12.3 [11.0, 14.1]	11.2 [9.8, 12.9]	0.056
FT3 at diagnosis (pmol/L)	2.4 [2.1, 3.0]	2.5 [1.9, 3.9]	0.9
FT4 normalized	78 (84.8%)	10 (50.0%)	0.002
FT3 normalized	39 (42.4%)	4 (20.0%)	0.078
FT4 change (pmol/L)	1.3 [0.6, 2.1]	2.3 [0.7, 3.8]	0.035
FT3 change (pmol/L)	1.3 [0.8, 2.3]	0.7 [0.2, 1.2]	0.039
Age (years)	10.5 [7.0, 14.0]	9.5 [5.0, 12.0]	0.14
HbA1c (%)	11.4 [10.3, 12.4]	12.0 [10.8, 12.8]	0.2
Glucose at Diagnosis (mg/dL)	456.0 [429.0, 511.0]	529.0 [517.0, 610.0]	<0.001

DKA, diabetic ketoacidosis; FT3, free triiodothyronine; FT4, free thyroxine; TSH, thyroid-stimulating hormone; Q1, first quartile; Q3, third quartile. Mild and moderate categories were merged for analysis. Categorical variables were compared using Fisher’s exact test; continuous variables using Wilcoxon rank-sum test (non-parametric). *p* < 0.05 was considered statistically significant.

## Data Availability

Data used for the analysis are available using the following link https://alexuuni-my.sharepoint.com/:x:/g/personal/ramy_ghazy_alexu_edu_eg/IQAKwhVDrGDFS5yKJG1LR4U2ATAFPpuXU04BjzJRC8EZPoQ?rtime=e7oB8Jhv3kg (accessed on 13 February 2026).

## Abbreviations

The following abbreviations are used in this manuscript:

aOR	Adjusted Odds Ratio
BMI	Body Mass Index
CI	Confidence Interval
DKA	Diabetic Ketoacidosis
ESS	Euthyroid Sick Syndrome
FT3	Free Triiodothyronine
FT4	Free Thyroxine
HbA1c	Hemoglobin A1c
ICU	Intensive Care Unit
IQR	Interquartile Range
NTI	Nonthyroidal Illness
Q1	First Quartile
Q3	Third Quartile
SD	Standard Deviation
T1DM	Type 1 Diabetes Mellitus
T3	Triiodothyronine
T4	Thyroxine
TSH	Thyroid-Stimulating Hormone
